# Evidence of Aquaporin 4 Regulation by Thyroid Hormone During Mouse Brain Development and in Cultured Human Glioblastoma Multiforme Cells

**DOI:** 10.3389/fnins.2019.00317

**Published:** 2019-04-04

**Authors:** Lucas E. S. Costa, José Clementino-Neto, Carmelita B. Mendes, Nayara H. Franzon, Eduardo de Oliveira Costa, Vivaldo Moura-Neto, Adriana Ximenes-da-Silva

**Affiliations:** ^1^Instituto de Ciências Biológicas e da Saúde, Universidade Federal de Alagoas, Maceió, Brazil; ^2^Instituto do Cérebro and Universidade Federal do Rio de Janeiro, Rio de Janeiro, Brazil

**Keywords:** aquaporin 4, thyroid hormone, brain development, GBM, brain tumor

## Abstract

Accumulating evidence indicates that thyroid function and the thyroid hormones L-thyroxine (T4) and L-triiodothyronine (T3) are important factors contributing to the improvement of various pathologies of the central nervous system, including stroke, and various types of cancer, including glioblastoma multiforme (GBM). Low levels of T3 are correlated with the poorest outcome of post-stroke brain function, as well as an increased migration and proliferation of GBM tumor cells. Thyroid hormones are known to stimulate maturation and brain development. Aquaporin 4 (AQP4) is a key factor mediating the cell swelling and edema that occurs during ischemic stroke, and plays a potential role in the migration and proliferation of GBM tumor cells. In this study, as a possible therapeutic target for GBM, we investigated the potential role of T3 in the expression of AQP4 during different stages of mouse brain development. Pregnant mice at gestational day 18, or young animals at postnatal days 27 and 57, received injection of T3 (1 μg/g) or NaOH (0.02 N vehicle). The brains of mice sacrificed on postnatal days 0, 30, and 60 were perfused with 4% paraformaldehyde and sections were prepared for immunohistochemistry of AQP4. AQP4 immunofluorescence was measured in the mouse brains and human GBM cell lines. We found that distribution of AQP4 was localized in astrocytes of the periventricular, subpial, and cerebral parenchyma. Newborn mice treated with T3 showed a significant decrease in AQP4 immunoreactivity followed by an increased expression at P30 and a subsequent stabilization of aquaporin levels in adulthood. All GBM cell lines examined exhibited significantly lower AQP4 expression than cultured astrocytes. T3 treatment significantly downregulated AQP4 in GBM-95 cells but did not influence the rate of GBM cell migration measured 24 h after treatment initiation. Collectively, our results showed that AQP4 expression is developmentally regulated by T3 in astrocytes of the cerebral cortex of newborn and young mice, and is discretely downregulated in GBM cells. These findings indicate that higher concentrations of T3 thyroid hormone would be more suitable for reducing AQP4 in GBM tumorigenic cells, thereby resulting in better outcomes regarding the reduction of brain tumor cell migration and proliferation.

## Introduction

Thyroid hormones play important roles during the development and maturation of the nervous system, being involved in the processes of myelination, cell growth, cell migration, in addition to their well-known metabolic effects (Oppenheimer et al., 1991; Trentin and Moura-Neto, 1995; Mullur et al., 2014).

Several studies have shown that the main water channel protein in the brain, aquaporin 4 (AQP4) also participates in important brain processes, including cell migration (Saadoun et al., 2005; Papadopoulos et al., 2008) and regulation of the flow of metabolites and ions (Ho et al., 2009) and that its expression can be regulated by the changes in metabolism (Deng et al., 2014), extracellular fluid volume, and tumorigenesis (Saadoun et al., 2002; Noell et al., 2012).

The aquaporins (AQPs) are a family of integral membrane carrier proteins that mediate bidirectional water transport across the membrane cells in response to an osmotic gradient. Currently, 14 members (AQP 0–13) of the AQP protein family have been identified and characterized in humans and rodents. The AQPs are structurally organized into tetramers within the cell membrane and each monomer acts as a pore for conducting water. Certain isoforms of AQPs may also mediate the transport of small solutes, such as glycerol, in addition to the transport of gasses (CO_2_, NH_3_, NO, and O_2_) and ions (K^+^ and Cl^-^) (Papadopoulos and Verkman, 2013).

Thyroid hormones are essential for brain development and metabolic homeostasis. Their deprivation during pregnancy, even if modest, causes abnormal cortical development and changes of synaptic function affecting fetal development (Goodman and Gilbert, 2007). A correlation between low levels of thyroid hormones and a predisposition to the emergence of diseases affecting the central nervous system (CNS) has been demonstrated. Thus, patients with low serum levels of the hormone L-triiodothyronine (T3) exhibit a greater predisposition to strokes (Jiang et al., 2017), and there also appears to be a correlation between the levels of thyroid hormones and certain types of astrocytomas (Ding et al., 2013; Xiong et al., 2018), such as glioblastoma multiforme (GBM).

Gliomas are tumors originated in glial cells, which are classified as astrocytomas, oligodendrogliomas, ependymomas, and glioblastomas. The GBM is a diffuse high-grade astrocytoma with high invasiveness and migration capacity, which makes it very difficult to treat and thus, reducing patients’ life expectancy to about 12 to 14 months after the diagnosis (Lacroix et al., 2001).

The expression of AQP4 is extremely correlated with the degree of severity of the astrocytoma (Warth et al., 2005; Zhao et al., 2012), tissue edema formation in the peritumoral region (Saadoun et al., 2002), increased cell migration (Saadoun et al., 2005; Papadopoulos et al., 2008) and disorganization of the characteristic arrangement of AQP4 in orthogonal arrays of particles (OAPS) in perivascular astrocytes endfeet (Noell et al., 2012).

These changes together, would contribute to the increase in brain swelling, rupture of the blood-brain barrier and disorganization of extracellular matrix (ECM) proteins, found in GBM tumors (Dubois et al., 2014).

Glioblastoma has also been reported to be a thyroid hormone-dependent tumor, in which these hormones would act promoting growth, migration, and development of tumor cells (Nauman et al., 2004; Davis et al., 2006; Sudha et al., 2017). Conversely, other studies have reported that, acting in non-genomic pathways thyroid hormone could reduce malignant cell proliferation and therefore, be a potential therapeutic agent in GBM (Martínez-Iglesias et al., 2009, 2016).

In astrocytes, T3 regulates protein expression in the ECM during brain development and the secretion of growth factors, which, in the cerebellum, act in an autocrine manner, inducing astrocyte proliferation, ECM reorganization, and cerebellar neuroblast proliferation, and are assumed to affect other pathways in the CNS via astrocytic cells (Trentin et al., 1995, 2001; Trentin, 2006).

Studies on the molecular mechanisms of thyroid hormone action and in the development and course of cancer have indicated a route of gene action, in which the pro-hormone thyroxine (T4) acts via integrin αvβ3, MAPK signaling, and ERK mitogen (1/2-protein kinase and extracellular signal-regulated kinase 1/2), mediated by phosphorylation of the thyroid β1 receptor (TRβ1), thereby inducing angiogenesis and tumor proliferation (Bergh et al., 2005). Although the effects of thyroid hormones on AQP expression, particularly in the CNS, are still largely unknown, current studies have shown T3 modulation of the expression of the liver mitochondrial isoform of aquaporin AQP8 in hypothyroid rats, indicating that T3 negatively regulates the AQP8 gene (Calamita et al., 2007).

In the present study, we aimed to evaluate whether the main water channel in astrocytes of the CNS (AQP4) could be regulated by T3 in normal brains at several stages of development (mice at P0 to P60) and also in brain glioma tumors.

The initial evaluation was carried out in cell culture using lines of human glioblastoma cells (GBM-11, GBM-95, and GBM-02), generously donated by Dr. Vivaldo Moura-Neto, as well as U-87 and SCC-4 [squamous cell carcinoma (SCC-4) of the tongue] cell lines. The effects on healthy cells were evaluated through the human HaCat keratinocyte cell line, and in a secondary culture of astrocytes derived from E16 mouse embryos. Finally, the effect of T3 on the expression of AQP4 and tumor cell mobility and invasiveness were analyzed in cell culture using a “scratch assay.”

## Materials and Methods

### Animals

Swiss mice at approximately the 18th day of pregnancy, provided by the Central Vivarium of the Federal University of Alagoas (BIOCEN-UFAL), were maintained in an air-conditioned room at 22°C and under a light-dark cycle of 12 h (lights on: 0700–1900). Pregnant mice at gestational day 18, or young animals on postnatal days 27 and 57, received injection of T3 (1 μg/g) or NaOH (0.02 N vehicle) during 3 consecutive days, intraperitoneally. The animals were anesthetized at postnatal days P0, P30, and P60 (*n* = 3–4 for each group) and then perfused with 0.9% NaCl followed by 4% paraformaldehyde, after which their brains were dissected and post-fixed in 4% paraformaldehyde at 4°C.

This study was carried out in accordance with the recommendations of the Brazilian guide for the care and use of laboratory animals and local ethics committee. The protocol was approved by Animal Ethics Committee from Federal University of Alagoas (approval number 25/2013).

### Immunohistochemistry

After post-fixation in 4% paraformaldehyde for 4 h, the brains were immersed in 30% sucrose solution at 4°C until subsequent preparation for microscopic analysis. Coronal section (40 μm) were cut using a cryostat (-20°C) and arranged on gelatinized slides. For immunohistochemical analysis, glial fibrillary acidic protein (GFAP) labeling was used to identify the location of AQP4, specifically in astrocytes. Briefly, sections were washed with phosphate-buffered saline (PBS) for 5 min (three times), immersed in 0.5% Triton X-100 in PBS solution (30 min), rinsed with PBS for 5 min (three times), and then blocked with 1% bovine serum albumin (BSA) for 90–120 min. Thereafter the sections were incubated overnight at 4°C with primary antibodies diluted in 1% BSA (anti-AQP4 1:200, Merck # AB3594; anti-GFAP 1:200, Dako #Z0334 ). The following day, after washing three times with PBS for 5 min, the sections were incubated with secondary antibodies (Alexa Fluor 448, Invitrogen #A11008 and Alexa Fluor 568 Invitrogen #A11004, 1:1000) diluted in 5% normal goat serum (1 h at room temperature), rinsed with PBS for 5 min (three times) and arranged on slides with PBS + glycerol solution (1:1). To evaluate the distribution of AQP4 in the brains of mice treated and non-treated with T3, the sections were observed under a fluorescence optical microscope (Nikon^TM^). Cells showing immunoreactivity for AQP4 were quantified using the ImageJ imaging program.

### Cell Culture

Human glioblastoma cells (GBM-95, GBM-02, and GBM-11) were kindly provided by Dr. Vivaldo Moura-Neto, and U87, HaCat, and SCC-4 cell lines were obtained from the American Type Culture Collection. These cells were cultured in Dulbecco’s modified Eagle’s medium (DMEM) F12 containing 10% fetal bovine serum (FBS), 10,000 U/mL penicillin, and 10,000 μg/mL streptomycin. Cultures were incubated at 37°C in a humidified atmosphere at 5% CO_2_/95% atmospheric air.

### E16 Astrocyte Secondary Culture

Pregnant Swiss mice, anesthetized with 100 mg/kg ketamine and 10 mg/kg xylazine, were submitted to cesarean surgery on the 16th embryonic day (E16). The uterus was placed in a Petri dish containing PBS and the embryos were removed. The brains were dissected and their cortices were placed in serum-free DMEM F12 culture medium for punching and cell dissociation. After centrifugation at 1500 rpm and 4°C, the supernatant was discarded and the pellet resuspended in serum medium for cell counting in a Neubauer chamber. The cells were plated in 25 mL bottles and the medium changed on alternate days to prevent neuronal growth. After 7 days, the cells were removed and plated again for treatment.

### Cell Treatment

After reaching confluency, the cells were treated with 50 nM T3 in serum-free medium or only with serum-free medium for 24 h. Control cells were treated with serum-containing medium. After 24 h, the conditioned medium of the T3-treated cells was withdrawn and maintained at -20°C.

### Immunocytochemistry

After washing with PBS, the cells were fixed in 4% paraformaldehyde, permeabilized with 0.3% Triton X-100, and blocked for non-specific binding with 5% BSA. Cells were incubated overnight in a refrigerator at 4°C in 0.3% blocking solution containing a polyclonal anti-AQP4 antibody (1:100) as described above. The following day, after washing with PBS, the cells were incubated with Alexa Fluor 546, Invitrogen # Z25304 anti-rabbit secondary antibody and/or Alexa 488-conjugated phalloidin, Invitrogen #A12379 (both 1:500). The nuclei were labeled with 1 mg/mL DAPI (4′,6-diamidino-2-phenylindole) and the coverslips glued with fluoromount. Photographs of cell were obtained at ×60 magnification.

### Scratch Assay

Cells were plated in six-well plates and maintained under the same culture conditions until they reached confluence, at which time they were crossed with two cross-shaped scratches, performed using a 10-μL pipette tip, and photographed under a ×20 objective lens. After treatment with 50 nM T3 for 24 h, the cells were photographed once again.

### Fluorescence Analysis

For quantification of fluorescence intensity, sections of mice brains were viewed at 10× using a fluorescence optical microscope (Nikon^TM^). Images captured by a camera were saved in .jpeg format and quantified using the ImageJ program. An average of 6–10 sections by animal was analyzed, corresponding to AP: -2.1; and -2.5 mm from Bregma. The images were opened and a ‘threshold’ was settled. After putting a dark background, fluorescent stained areas for AQP4 were selected, and measured. The fluorescence intensities of the total cerebral cortex were averaged for each section and by group. AQP4 fluorescence intensity in cell lines HaCat, GBM 95, SCC-4, U-87 and secondary culture of astrocytes was quantified based on area of selected cell vs. mean fluorescence of background readings.

### Statistical Analysis

Fluorescence quantification was performed based on the integrated densities of selected areas, using the ImageJ imaging program, and significance analysis of the differences between the conditions was performed with a one-way ANOVA. A Bonferroni post-test was used to compare the integrated densities between different treatments of the same cell type. Analysis of the scratch assay results was performed by comparing the means of the measures of the risk area at time zero and after 24 h of treatment. Statistical analyses were performed with GraphPad Prism 6.0 (GraphPad Software, Inc., San Diego, CA, United States).

## Results

### Immunohistochemistry

**FIGURE 1:**
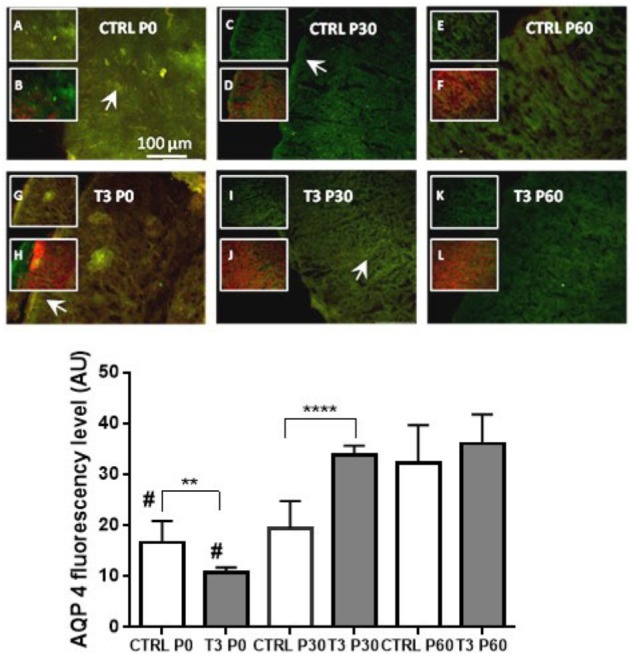
**(Upper)** Aquaporin 4 (AQP4) distribution in the cerebral cortex of mice at postnatal days 0, 30, and 60 in control (CTRL) and T3-injected animals. Insets depict AQP4 immunoreactivity (green: A, C, E, G, I, and K) or merged GFAP (red) colocalization (B, D, F, H, J, and L). Immunoreactivity of AQP4 showed polarization to perivascular astrocytic end feet surrounding the cerebral microvessels (arrowheads in CTRL P0 and T3 P30) or in subpial astrocytes as depicted in CTRL P30 and T3 P0 slices. T3 injection caused an increase in AQP4 immunoreactivity with developmental progression. **(Lower)** AQP4 fluorescence levels in coronal sections of total brains. Mice treated with T3 showed higher levels of AQP4 from 30 to 60 days of age. *N* = 3 or 4 for each group; fluorescence analysis from 6 to 10 coronal sections of 40-μm thickness. Student’s *t*-test; ^∗∗^*P* < 0.01; ^∗∗∗∗^*P* < 0.0001; #*P* < 0.0001 one-way ANOVA followed by Tukey’s test (CTRL P0 vs. CTRL P60; T3 P0 vs. T3 P30 and T3 P60).

Immunofluorescence analysis was performed on serial coronal sections (*n* = 3 or 4 animals, 6 to 10 sections). The results, shown in Figure 1, indicate that, relative to the control group, the treatment of mice with 1 μg/g T3 (E18–E20) reduced the distribution of AQP4 in the cerebral cortex on postnatal day P0 (Student’s *t*-test: *P* < 0.02). However, T3-treated young mice showed a significant increase in AQP4 at postnatal day 30 (Student’s *t*-test: *P* < 0.0001), which remained stable until the 60th postnatal day (P60). In the control animals, there was a significantly increased variation in AQP4 over the three postnatal developmental stages (P0, P30, and P60), indicating that T3 treatment contributes to the increased distribution of AQP4 in the cerebral cortex (one-way ANOVA: *P* < 0.0001).

Figure 1 shows the fluorescence microscopic images depicting the distribution of AQP4. A diffuse fluorescence can be observed in the entire cerebral cortex, with a more pronounced distribution of AQP4 being observed on the surface of astrocytic end feet surrounding the cerebral capillaries and the cortical surface near the pia mater, as has previously been described in the literature. Treatment with T3 resulted in a biphasic expression of AQP4 in the cerebral cortex of mice, with a decrease in expression being observed at the beginning of postnatal life (P0: *P* < 0.01), followed by an increase in expression at 30 days of life, relative to the control group.

### *In vitro* Studies

**FIGURE 2:**
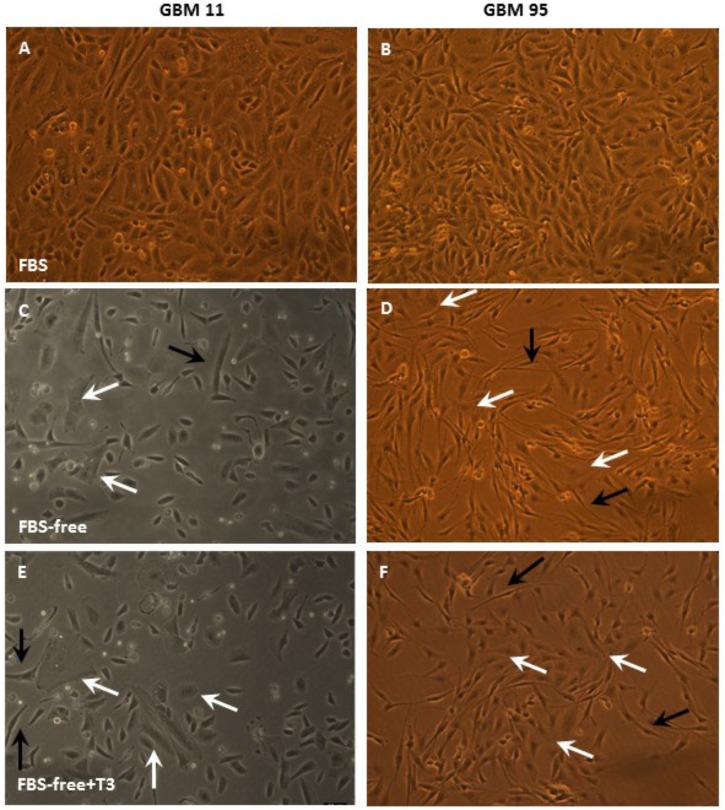
Phase-contrast microscopic images of glioblastoma multiforme GBM-11 and GBM-95 cells cultured in medium containing 10% fetal bovine serum (FBS), medium without serum (FBS-free), and treated with T3 in medium without serum (FBS-free+T3). GBM tumor cells treated with T3 showed large cytoplasmic volume (**E,F**, white arrows) and processes (**E,F**, black arrows) when compared with those maintained in FBS-free medium **(C,D)**. GBM-11 and GBM-95 cells maintained in serum-containing medium **(A,B)**.

**FIGURE 3:**
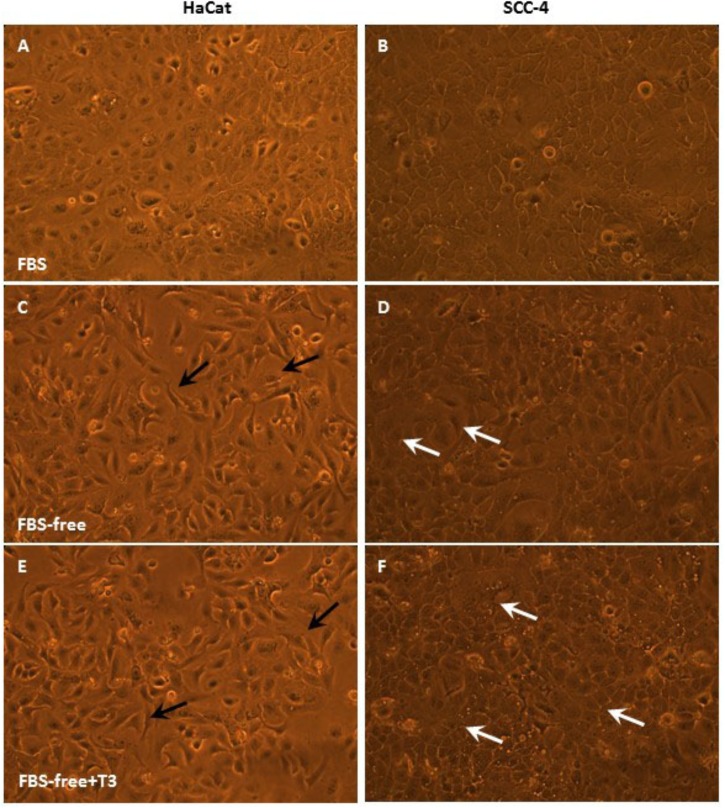
Phase-contrast microscopic images of HaCat and SCC-4 cells in medium containing 10% FBS, medium without serum (FBS-free), and treated with T3 in medium without serum (FBS-free+T3). In the SCC-4 line, cells grown in T3-treated and FBS-free medium showed more disorganized cell–cell contacts **(C,D)** compared with those of cells maintained in serum-containing medium **(A,B)**. White arrows indicate large cytoplasmic volume **(D,F)** and black arrows cytoplasmic processes **(C,E)**.

Figure 2, 3 depict phase contrast microscopic images of GBM-11 and GBM-95 (Figure 2), HaCat and SCC-4 (Figure 3) cells grown under three different conditions: DMEM-F12 culture medium supplemented with 10% FBS, the same medium without serum supplementation (FBS-free), and T3 supplemented FBS-free medium (FBS-free + T3). Analyses of the images indicated that treatment of cells with T3 in FBS-free medium caused morphological changes in all the studied cell lines, leading to an increase in cytoplasmic volume and cellular processes. The images of GBM-95 and GBM-11 cells indicate that treatment with T3 in the FBS-free medium caused protoplasmic changes in the cells, producing a more elongated morphology (C, D, E, and F) relative to cells maintained in FBS medium (A and B). Moreover, T3 treatment also led to an increased number of cells with increased cytoplasmic volume, compared with cells maintained in FBS-free medium. HaCat cells treated with T3 and those maintained in FBS-free also exhibited a more elongated protoplasmic morphology (C, D, E, and F), relative to cells maintained in FBS (A and B), whereas no difference was observed between the T3-treated group and the cells maintained in FBS-free medium. In the SCC-4 cell line, T3-treated cells and those maintained in FBS-free medium were found to be more disorganized (C and D), less delimited, and more heterogeneous in shape than cells maintained in FBS medium (A and B).

### Immunofluorescence – Cell Culture

**FIGURE 4:**
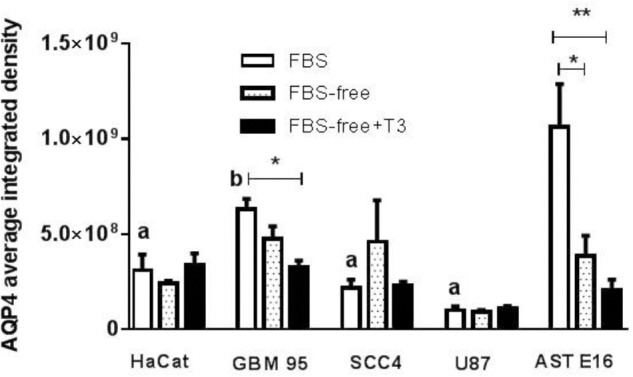
Fluorescence analysis for AQP4 in HaCat, GBM-95, SCC-4, and U-87 cell lines and a secondary culture of astrocytes (AST E16). The integrated density is the sum of the values of the pixels in the area considered fluorescent. T3 treatment significantly downregulated AQP4 in GBM-95 cells maintained in FBS. Among all the cell lines examined, astrocytes exhibited the highest expression of AQP4. T3 treatment in FBS-free medium reduced AQP4 expression in astrocytes. HaCat, SCC-4, and U87 cell lines showed no change in AQP4 expression. Cells were cultured in medium containing 10% FBS, medium without serum (FBS-free), and treated with T3 in medium without serum (FBS-free+T3). ^∗^*P* < 0.05, ^∗∗^*P* < 0.01, ^∗∗∗^*P* < 0.001; and (a) *P* < 0.001; (b) *P* < 0.05 are related to differences between the tumor cells in FBS medium vs. secondary culture of Ast E16 in FBS.

**FIGURE 5:**
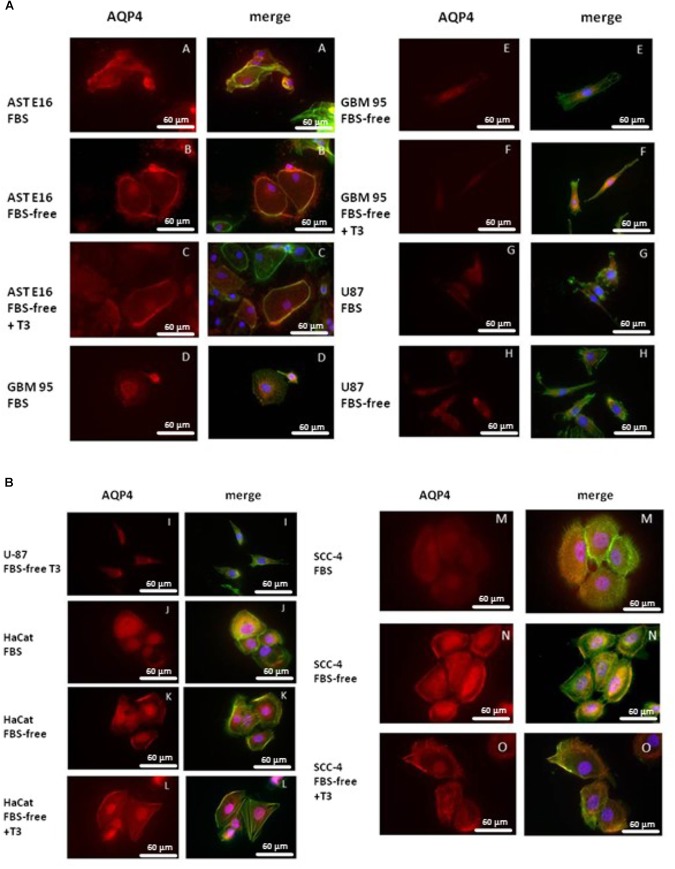
**(A)** Immunofluorescence staining for AQP4 (red) and phalloidin (green) or DAPI (blue). T3 treatment (50 mM) negatively regulated AQP4 expression only in GBM-95 cells when compared to AQP4 expression in cells maintained in FBS, as shown in Figure 6. Cells were cultured in medium containing 10% FBS, medium without serum (FBS-free), and treated with T3 in medium without serum (FBS-free+T3). **(B)** Immunofluorescence staining for AQP4 (red) and phalloidin (green) or DAPI (blue). HaCat, SCC-4, and U87 cell lines treated with T3 (50 mM) showed no change in AQP4 expression.

Figure 4 presents immunofluorescence results obtained from an analysis of the integrated densities of images using the Prism program. Figure 5A,B show the results obtained using immunofluorescent markers for AQP4, red (left) and the merged images of AQP4, phalloidin, and DAPI (right). As shown in Figure 5A (D, E, and F), treatment with T3 negatively regulated AQP4 expression in the GBM-95 glioblastoma culture only when compared with AQP4 expression in cells maintained in FBS. Observation of a secondary culture of E16 astrocytes showed that the two groups maintained in FBS-free medium exhibited a significant difference in the expression of AQP4 relative to the group maintained with FBS only, although no statistically significant difference was observed between the FBS-free groups, suggesting that not only the T3 treatment but also the FBS-free conditions may have negatively regulated AQP4 expression, as shown in Figure 5A (A, B, and C). Analysis of SCC-4 cells indicated that the T3 treatment negatively regulates AQP4 expression compared with the cells maintained in FBS-free medium; however, this difference was not statistically significant according to Figure 4, 5B (M, N, and O). Figure 4, 5 indicate that there were no significant differences in AQP4 expression in U-87 and HaCat cells related to T3 treatment or in response to the different culture media. However, as shown in Figure 4, all the cell lines examined showed significantly lower AQP4 fluorescence staining compared with the secondary cultures of astrocytes. Thus, our initial studies indicated that, compared with the treatment control (FBS-free medium), T3 treatment had no significant influence on the expression of AQP4 in any of the cell types examined.

### Scratch Assay

**FIGURE 6:**
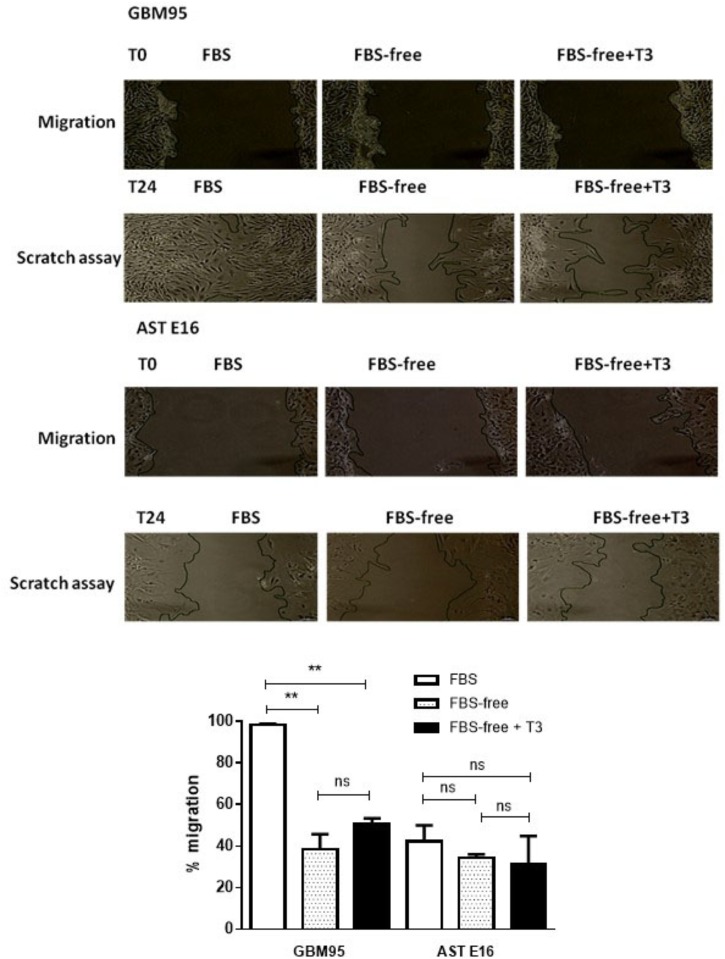
**(Upper)** Analysis of cell migration in GBM-95 cells and cultured E16 astrocyte cells. The migration and invasion of the GBM cell line was not significantly altered by treatment with T3, whereas the migration of cultured astrocytes was significantly decreased by 24.47% in response to treatment with T3. **(Lower)** Cell migration assay of GBM-95 cells and cultured E16 astrocytes treated with T3. Assay measurements were performed at two different time points: T0, represents the beginning of the treatment and T24 represents 24 h after the start of treatment. Cells were cultured in medium containing 10% FBS, medium without serum (FBS-free), and treated with T3 in medium without serum (FBS-free+T3). The images were analyzed by measuring the reduction in scratch area (^∗∗∗^*P* < 0.001: one-way ANOVA).

Figure 6 shows the migration of cells of the GBM-95 line and the secondary culture of E16 astrocytes under our three treatment conditions: FBS, FBS-free, and FBS-free + T3. In this study, we used the FBS-free medium to reduce cell proliferation. For each treatment, we subjected the crossed out area to statistical analysis. Time T0 represents the beginning of treatment and T24 represents cell migration after 24 h.

We found that after 24 h, there was no statistically significant difference between cells grown in FBS-free +T3 and control (FBS-free) media. The total scratched area of GBM95 in FBS revels rather cell proliferation, than only cell migration (*P* < 0.01). In control astrocytes (AST E16) treated with both FBS and FBS-free medium, unexpectedly cell migration difference was not observed, although it was noted a tendency to decreased in cell migration.

## Discussion

In this study, we sought to determine whether the administration of T3 has a regulatory effect on the expression of AQP4 in astrocytes during the normal development of the CNS and in cells derived from glioblastomas, the major type of brain tumor. The results indicated that the expression of AQP4 in the mouse brain varies according to the developmental phase of the nervous system and that the thyroid hormone T3 modulates this expression during the different phases. In addition, experiments performed on a culture of normal astrocytes and glioma cells showed a potential effect of T3 on the expression of AQP4.

The lower expression of AQP4 at the onset of postnatal life observed in the control animals of our study could be related to a reduction in water volume in the brain that occurs during development. In mice, the decreased brain water content is more marked from postnatal day 14 to 21, corresponding to 84 and 79% of the adult brain water content, respectively. In this regard, it has previously been demonstrated that the decrease in postnatal brain water content is delayed in AQP4-null mice (Li et al., 2013). Interestingly, a higher expression of AQP4 during the period between postnatal days 7 and 14 has been found to precede a marked reduction of brain water volume in wild-type mice, indicating that higher expression AQP4 is pivotal for reduced CSF during the early postnatal weeks (Wen et al., 1999; Hsu et al., 2011; Li et al., 2013).

Fallier-Becker et al. (2014) demonstrated that during the development of the CNS in the P1–P3 stage, the distribution of AQP4 immunoreactivity became increasingly restricted to the subpial and perivascular end feet. The results of the present study are consistent with those of the aforementioned studies, in that we observed a lower level of AQP4 in the P0 control group relative to that in the adult animals, and that these difference were more marked in animals treated with T3, which exhibit a significant reduction of AQP4 at P0, and an increased brain expression of this protein at P30 and P60.

During pregnancy, there is limited T3 transport through the placenta, and T4 produced in the placenta is the main source of T3 for the developing fetal brain. In the brain, T4 is converted to T3 by the enzyme deiodinase 2 (D2) present in astrocytes. Experiments carried out by Bárez-López and Guadaño-Ferraz (2017) and Bárez-López et al. (2018) using mice whose mothers had received thyroid hormone T4 in their drinking water during the E12–E18 embryonic phase revealed an increase in T3 and a reduction in T4 levels in maternal blood, whereas pups in the perinatal phase (P1) showed low levels of T4 and no change in T3 levels in the blood and brain. In the present study, pregnant females received subcutaneous injections of T3 for three consecutive days (E18–E20), and although thyroid hormone levels were not measured, it can be assumed that a similar alteration in the levels of these hormones occurred in the P0 animals, since during fetal life the transport of T3 through the placenta is limited. Thus, we conclude that the contribution of T4 in the animals of the P0 group would be decreased, which may have led to an increase in the activity of the D2 enzyme in the fetal brain to compensate for the fall of T4, thereby ensuring local T3 formation in the fetal brain.

It has recently been demonstrated in mice that T4, mostly of maternal origin, is transported via the cerebrospinal fluid reaching the fetal brain where it is converted into T3 through the astrocytic D2 enzyme present in the blood-brain barrier (meninges and choroid plexus) and lateral ventricles (Bárez-López et al., 2018). From the cerebrospinal fluid, the T3 thus produced can disperse throughout the brain and access the cells to exert its action. D2 enzyme activity decreases around P3 and was not detected in the meninges, choroid plexus, or lateral ventricles of adult rodents. Assuming that T3 injection in the mothers caused a decrease in T4 in fetal blood of the animals examined in the present study, there would be an increase in the activity of D2 enzyme in astrocytes to compensate for the decrease in T4 to ensure the maintenance of T3 levels in the brain. Concomitantly, a decrease in the action of the enzyme D3 in neurons would ensure the reduction in the metabolism of T3 to rT3 and T2, thereby ensuring the supply of T3 to the brain. Accordingly, T3 would act on astrocytes, leading to a reduction in AQP4 expression, as observed in the P0 animals examined in this study.

It is known that thyroid hormones play a role in the reduction of AQP4 expression through non-genomic pathways by protein kinase C (PKC) activation (Sadana et al., 2015). The PKC pathway when activated leads to the phosphorylation of AQP4 (residue Ser 180), causing a reduction of this water channel (Zelenina et al., 2002). Reduced AQP4 expression found in the brains of P0 T3-treated animals in our study could have been mediated by the conversion of circulating T4 to T3 in the astrocytes, promoting a reduction in AQP4 via PKC activation. Subcutaneous injections of T3 in young and adult animals in our study could be supposed to promote an increase of T3 supply to the brain, via the MCT8 transporter present in the blood–brain barrier increasing neuronal D3 activity to regulate cerebral T3 levels (Roberts et al., 2008). Decreased activity of T3 in the astrocytes could reduce AQP4 phosphorylation, thereby increasing the expression of this protein in the brain of young mice. Supporting this, studies have shown that adult Dio3KO mice exhibit an increase in T3 concentration, leading to a state of central hypothyroidism and decreased expression of T3-regulated genes (Hernandez et al., 2010, 2012), which is consistent with the data obtained for adult P60 animals in the present study, in which we found no significant difference in the expression of AQP4 between the control and T3-treated animals.

We assume that the effects of T3 administration on the expression of AQP4 observed in the present study are not attributable to the possible toxic effects of T3 on the brain or thyroid, given that the experimental protocols used to induce hyperthyroidism in animals involve administration of this hormone for approximately 14 consecutive days and at a dosage of 250 μg/kg (Drover and Agellon, 2004), which is considerably higher than the used in the present study.

In the present study, we have also examined the effects of T3 on the expression of AQP4 in cultured human glioblastoma cells. We accordingly found that all the cell lines examined showed significantly lower AQP4 expression compared with that observed in the secondary culture astrocytes, thereby confirming the previously established principal site of AQP4 in this cell type. Nevertheless, a significant effect on AQP4 expression was not observed under all conditions upon treatment with T3, relative to the treatment control, although we did observe a tendency for a reduction in AQP4 expression in GBM-95 cells and normal astrocytes.

Evaluation of the effects of T3 on cell migration after 24 h of treatment showed that in both normal astrocytes and GBM95 cells there was no difference in the migratory process. The reduction found in GBM95 FBS-free and GBM95 FBS-free + T3 medium was probably attributed to cell proliferation decrease, more than cell migration, as a result of the absence of FBS in the two treated groups (Figure 6). Our studies examining astrocyte and glioblastoma cell cultures indicated a tendency for T3 to negatively regulate the expression of AQP4 under the conditions described above (Figure 4), which did not influence the cellular migration process.

Preliminary data on cell cultures indicated a discrete down-regulation of AQP4 expression in GBM-95, SCC-4, and astrocyte secondary culture cells subjected to the serum-free medium and T3 treatments. Previous studies on the effect of thyroid hormones on the development, migration, and growth of gliomas, in particular GBM, have yielded contradictory data, indicating either a protective action of these hormones on tumor development or a proliferative action in the development of gliomas (Sudha et al., 2017). In this regard, some epidemiological studies have provided evidence of the role of thyroid hormones in promoting tumor cell proliferation, arguing that several different types of tumors exhibit increased growth in patients with a history of hyperthyroidism (Moeller and Führer, 2013). For example, it has been found that patients with a history of hyperthyroidism are more likely to develop ovarian cancer, whereas patients with pancreatic cancer have a two-fold higher risk of developing this type of tumor when they have hyperthyroidism. Notably, these epidemiological studies report an increased probability of enhanced proliferation of cancer cells under conditions of a pathological increase in the release of thyroid hormones (hyperthyroidism).

Studies by Davis et al. (2006) have highlighted the role of thyroid hormones in the growth of gliomas. These hormones have been shown to act as growth factors on gliomas, acting non-genomically via the αVβ3 receptor, to promote cell growth. There is evidence that T3 binding to the S1 site of the αVβ3 receptor activates PI3K (lipid kinase) and increases cellular invasiveness and metastasis, whereas the binding of T4 to the S2 site of this ERK1/2 active receptor alters FGF2 expression and promotes angiogenesis, with both these pathways favoring tumor growth. In contrast, more recent studies have indicated the more prominent action of T4 in tumorigenesis, relative to T3. Using a deaminated T4 derivative, tetraiodothyroacetic acid, linked to nanoparticles as an anticancer agent in the treatment of human GBM U87MG xenografts cells in immunodeficient mice, Sudha et al. (2017) demonstrated a reduction in tumor cell density (of up to 80%) and tumor necrosis caused by the T4 derivative possibly acting by inhibitory effect of extracellular domains of integrin αvβ3 T4 receptor. Other studies appear to highlight the greater activity of T4 in the processes of tumorigenesis, when compared to the direct effects of T3.

## Conclusion

In conclusion, in this study we demonstrated that T3 induces morphological alterations in normal and GBM-95, GBM-11, HaCat, and SCC-4 cells. Moreover, T3 treatment resulted in a positive regulation of AQP4 expression in the brain of mice at three different stages of brain development: immediately after birth and at 30 and 60 days of age. Astrocyte and glioblastoma cell culture studies revealed the tendency of T3 to negatively regulate the expression of AQP4 under the experimental conditions described herein, without significantly disrupting cellular migration. Further studies should be carried out to confirm the present results regarding the effects of T3 on the decreased expression of AQP4 and its relationship with tumor cell migration, and thereby unravel the possible role of the biologically active form of this hormone in altering the migration pattern of these cells.

## Data Availability

The datasets for this manuscript are not publicly available because the data are the results of research projects that are being submitted for the first time for publication. Requests to access the datasets should be directed to ximenes.adri@gmail.com.

## Author Contributions

AX-d-S and VM-N conception and design of the experiments. LC, JC-N, CM, NF, and EC collected the data. AX-d-S, LC, JC-N, and CM analyzed and interpreted the data. AX-d-S, JC-N, and CM drafted the article and critically revised for important intellectual content. All authors approved the final version of the manuscript.

## Conflict of Interest Statement

The authors declare that the research was conducted in the absence of any commercial or financial relationships that could be construed as a potential conflict of interest.
